# All-reflective tethered capsule endoscope for multimodal optical coherence tomography in the esophagus

**DOI:** 10.1117/1.JBO.29.9.096003

**Published:** 2024-09-19

**Authors:** Xavier Attendu, Paul R. Bloemen, Niels H. Kind, Dirk J. Faber, Daniel M. de Bruin, Caroline Boudoux, Ton G. van Leeuwen

**Affiliations:** a University of Amsterdam, Amsterdam University Medical Center, Department of Biomedical Engineering and Physics, Amsterdam, The Netherlands; b Polytechnique Montréal, Centre d’Optique Photonique et Lasers, Department of Engineering Physics, Montréal, Québec, Canada; c University of Amsterdam, Amsterdam University Medical Center, Department of Development and Innovation of Medical Technologies, Amsterdam, The Netherlands

**Keywords:** optical coherence tomography, endoscopy, tethered capsule endoscope, multimodal imaging, fiber optics

## Abstract

**Significance:**

Esophageal cancer is becoming increasingly prevalent in Western countries. Early detection is crucial for effective treatment. Multimodal imaging combining optical coherence tomography (OCT) with complementary optical imaging techniques may provide enhanced diagnostic capabilities by simultaneously assessing tissue morphology and biochemical content.

**Aim:**

We aim to develop a tethered capsule endoscope (TCE) that can accommodate a variety of point-scanning techniques in addition to OCT without requiring design iterations on the optical or mechanical design.

**Approach:**

We propose a TCE utilizing exclusively reflective optics to focus and steer light from and to a double-clad fiber. Specifically, we use an ellipsoidal mirror to achieve finite conjugation between the fiber tip and the imaging plane.

**Results:**

We demonstrate a functional all-reflective TCE. We first detail the design, fabrication, and assembly steps required to obtain such a device. We then characterize its performance and demonstrate combined OCT at 1300 nm and visible spectroscopic imaging in the 500- to 700-nm range. Finally, we discuss the advantages and limitations of the proposed design.

**Conclusions:**

An all-reflective TCE is feasible and allows for achromatic high-quality imaging. Such a device could be utilized as a platform for testing various combinations of modalities to identify the optimal candidates without requiring design iterations.

## 1 Introduction

Esophageal adenocarcinoma (EAC), a histological subtype of esophageal cancer (EC), has become a growing concern in Western countries due to rapidly rising incidence rates in recent decades.^1^^,^^2^ As a result of this trend and of the largely asymptomatic nature of the disease leading to late diagnosis, EAC is expected to become a significant clinical and economic burden in the near future.^3^ Currently, surveillance and diagnosis of EC primarily rely on white light endoscopy (WLE) and random biopsies. However, this approach is limited in its ability to diagnose EC early as WLE cannot detect the subtle morpho-chemical changes associated with pre-cancerous lesions,^4^^,^^5^ such as variations in nuclear shape and size, nuclear pleomorphism, changes in glandular architecture, or neovascularization.^6^^,^^7^ Moreover, random biopsies only provide low sampling densities and a corresponding low probability of catching localized pre-cancerous lesions.^8^ Beyond the low sensitivity for early-stage detection, screening or surveillance in high-risk groups is further hindered by the high cost of endoscopic procedures, which typically require sedation and specialized personnel and equipment.^9^

Optical coherence tomography (OCT) has demonstrated significant potential for early detection of EC. It overcomes many limitations of the current practices by providing non-invasive, depth-resolved, microscopic imaging of the esophagus. In addition, its non-invasive nature and high imaging speed enable comprehensive imaging of the entire organ, increasing the likelihood of detecting localized (pre-)cancerous lesions. Previous studies have shown that OCT may be implemented to detect or differentiate various tissue types and conditions such as Barrett’s esophagus, low- and high-grade dysplasia, and specialized intestinal metaplasia.^10^^–^^13^ Furthermore, it has been demonstrated that OCT endoscopes, such as tethered capsule endoscopes (TCEs) or catheter probes with inflatable balloons, may be implemented without the need for sedation or specialized medical personnel, opening the door for potential screening applications.^14^^,^^15^

Despite promising results, OCT alone has yet to demonstrate sufficient performance to replace the standard practice of WLE and random biopsies. One approach to enhance the diagnostic capabilities of standard OCT is to combine it with other modalities that provide complementary information or additional functionality. Previously, systems for esophageal imaging have combined OCT with autofluorescence imaging,^16^ fluorescence imaging with an exogenous dye,^17^^–^^19^ color imaging,^20^ and laser marking.^21^^,^^22^ Other modalities have also been combined with OCT in endoscopic systems developed for other organs and pathologies. Such combinations include fluorescence lifetime imaging,^23^^–^^25^ near-infrared (NIR) fluorescence,^26^ diffuse reflectance spectroscopy,^27^ multispectral reflectance imaging,^28^ and photoacoustic imaging.^29^ The technical feasibility of such multimodal endoscopic systems has been demonstrated and could, in principle, be adapted for esophageal imaging. However, a key question remains: Which combination provides optimal sensitivity and specificity for the early detection of EC? Providing a reliable answer to this question inevitably implies *in vivo* imaging with various multimodal systems. The development and implementation of these systems present many technical challenges, such as optical or mechanical design iterations to accommodate the selected modalities. In particular, adapting the endoscopic probes is costly and labor-intensive and may require repeated clinical certification. As such, there is great potential value in the development of an imaging platform based on endoscopic probes capable of handling different optical modalities with minimal to no alteration.

To this effect, we herein present the first all-reflective tethered capsule endoscope (RTCE) designed to enable highly multimodal optical coherence tomography in the esophagus. The proposed device relies on two critical features for effective multimodal operation. The first is the use of double-clad fiber (DCF) and a double-clad fiber coupler (DCFC), which have been demonstrated as effective solutions for combining OCT with various modalities.^30^ Utilizing a single fiber for illumination and collection of multiple modalities guarantees pixel-wise co-registration, thus simplifying data acquisition and analysis. The second key feature is the exclusive use of reflective optics to achieve finite conjugation between the delivery fiber tip and the imaging plane. This design allows for achromatic and back reflection-free imaging within the limits of the mirror reflectance and the fiber transmission capabilities. In this paper, we first present the design, fabrication, and assembly steps necessary to realize the tethered capsule, as well as the tools developed for fine-tuning the internal alignment. We then characterize the optical performance of the complete device and demonstrate preliminary multimodal imaging by combining OCT with visible (VIS) reflectance spectroscopy over the 500- to 700-nm range. Finally, we discuss the proposed design’s limitations and future improvements.

## 2 Methods

**Fig. 1:**
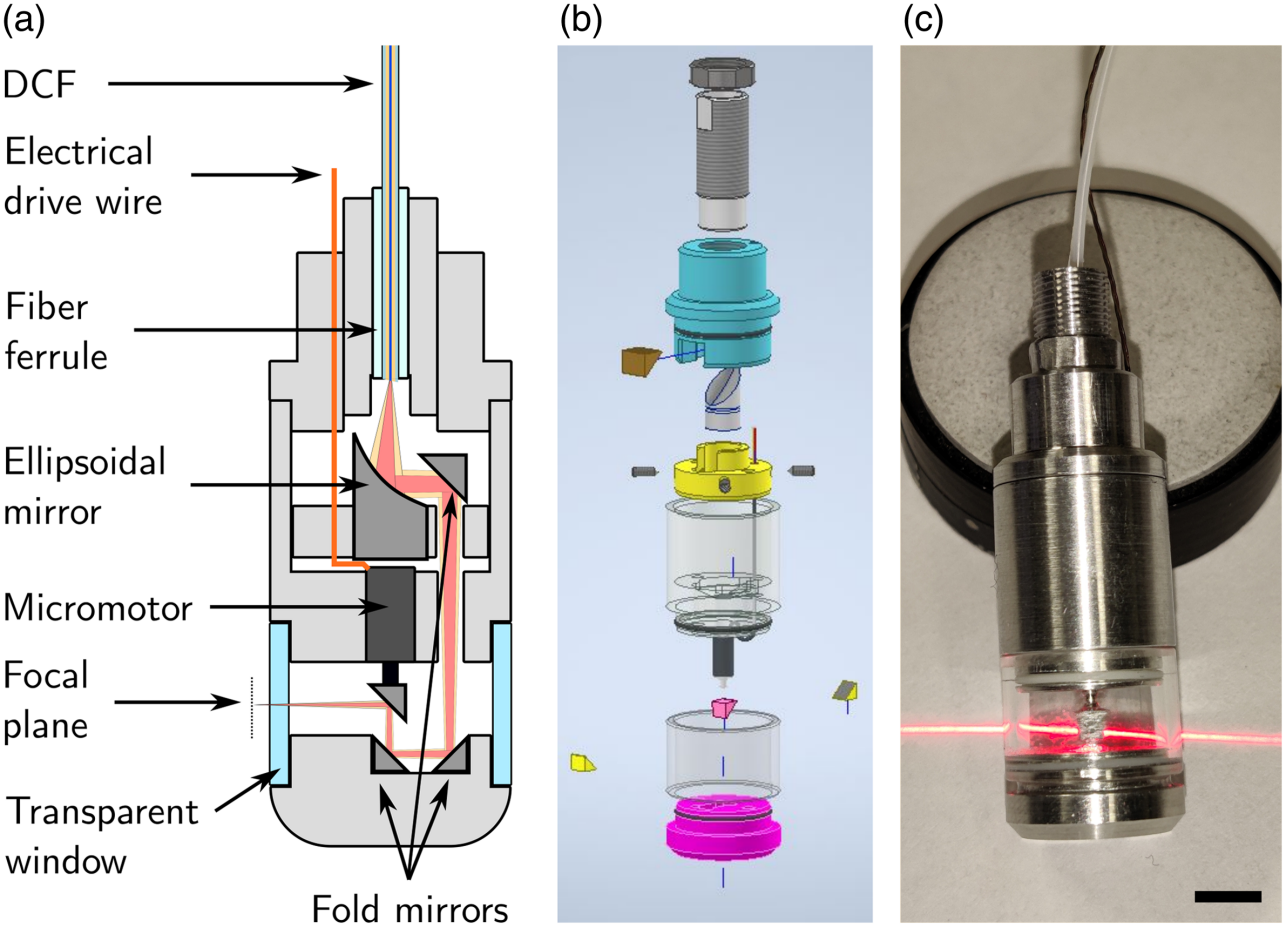
All-reflective tethered capsule endoscope. (a) Schematic representation of the reflective TCE. The red and yellow light beams represent the illumination from the core and inner cladding of the DCF, respectively. Schematic diagram not to scale. (b) Exploded view of the 3D model. (c) Photograph of the assembled tethered capsule endoscope with a red guide laser. The non-orthogonal orientation of the output beam is discussed in further detail in Sec. 2.3.2. The scale bar is 5 mm.

Figure 1 shows the proposed design for an all-reflective capsule endoscope, along with a photo of the assembled device. In line with previously published TCE designs implemented *in vivo*,^9^^,^^15^^,^^17^^,^^31^^,^^32^ we designed our capsule to have an outer diameter of 13 mm. In the prototype presented here, the total length is 35 mm (stiff length excluding the tether comprising the optical fiber and the electrical drive cable). Light delivery is performed using a DCF, allowing for various forms of multimodal imaging. The critical component differentiating this design from others is the ellipsoidal mirror (EM), which is used to achieve finite conjugation between the fiber tip and the focal plane. Precise design and alignment of the EM combined with beam folding allow for the positioning of the focal plane ∼500  μm outside the capsule, as illustrated in Fig. 1(a). The use of the EM, in particular, and reflective optics, in general, makes the assembly extremely sensitive to alignment errors.^33^ As such, the tolerances applied to the mechanical design were extremely high (∼10  μm for critical parts) to ensure the proper positioning of all components. Moreover, the optical alignment was carried out in steps to ensure optimal operation after adding each sub-assembly. Finally, beam scanning uses an inverted micromotor to avoid a shadow associated with the electrical drive wire, inspired by a previous design by Lopez-Marín et al.^34^

### 2.1 Optical Design

**Fig. 2:**
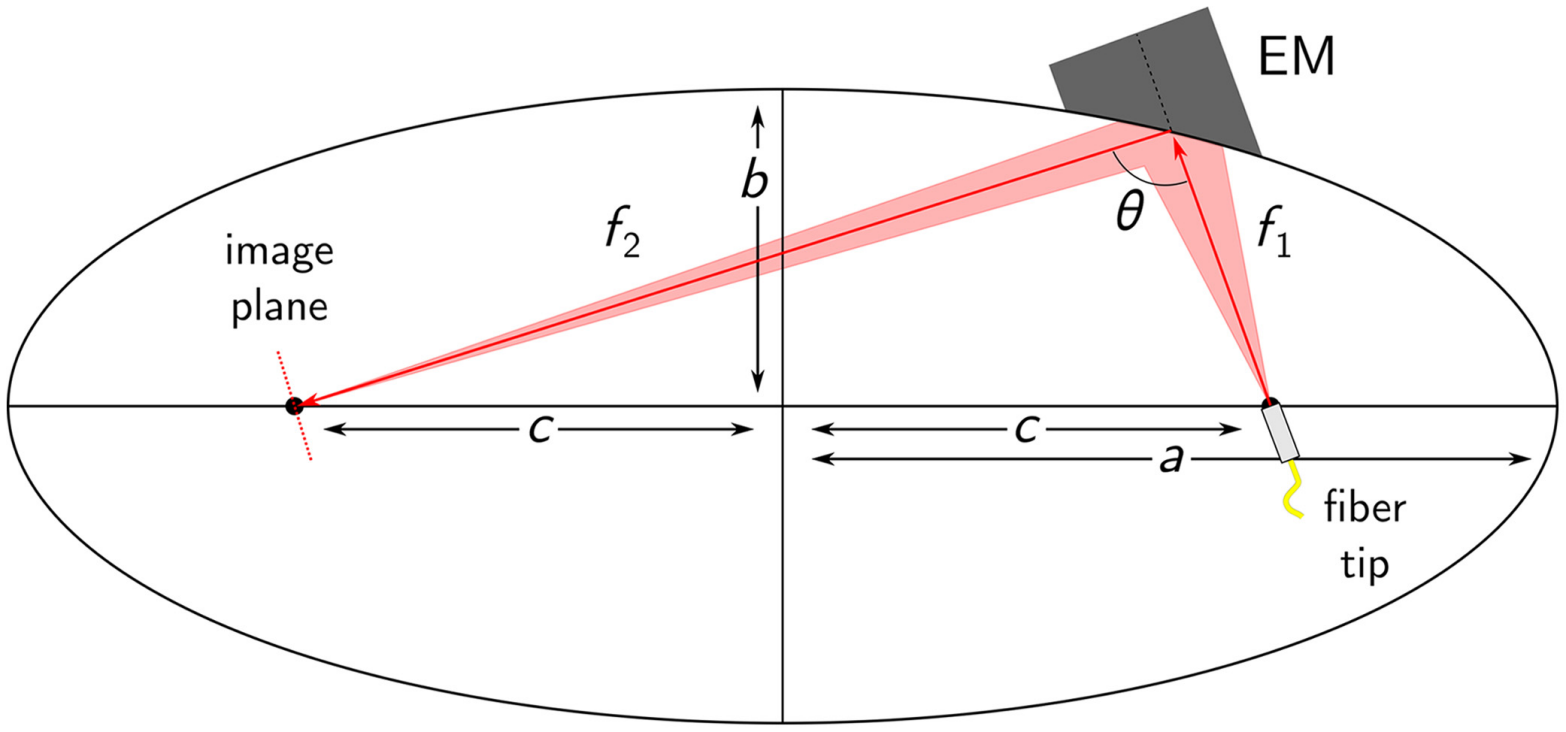
Geometrical representation of the elliptical section of the ellipsoidal surface. Variables $f_{1}$ and $f_{2}$ represent the two focal lengths, a and b are the lengths of the semi-axes, c is the distance from the origin to the foci (black dots), and θ is the deviation angle of the center ray.

The optical design of the proposed capsule is centered around a single, off-axis ellipsoidal mirror to achieve finite conjugation between the fiber tip and the focal plane located 0.5 mm outside the capsule surface. The EM surface is an ellipsoid of revolution, also called a spheroid, and is described in Cartesian coordinates as

$$\frac{x^{2}}{a^{2}}+\frac{y^{2}}{b^{2}}+\frac{z^{2}}{b^{2}}=1,$$ (1)

where a and b are the semi-axes of the elliptical cross-section along the XZ or the YZ plane, respectively. This cross-section, depicted in Fig. 2, can be rotated along the long semi-axis of length a to form the full ellipsoidal surface. Spheroids have the property of having two foci, where any ray emitted from one focal point will be reflected to the other, thereby achieving finite conjugation. For our application, this implies positioning the fiber at one focal point such that all emitted rays are imaged (i.e., focused) onto the second one, located at the sample plane.

Ellipses have the additional property that the lengths of the segments connecting the foci to any point on the ellipse must add to 2a, as described in Eq. (2). The foci are positioned at coordinates (0,±c), where c is given by Eq. (3)

$$f_{1}+f_{2}=2a,$$ (2)

$$c=\sqrt{a^{2}-b^{2}}.$$ (3)

Based on Eqs. (1)–(3), it is possible to fully define the ellipsoidal surface for a target pair of focal lengths and center ray deviation angle (θ). In the case of the TCE, these focal lengths are selected to satisfy three requirements. The first requirement is a given value for the numerical aperture (NA) in image space of the focused light. For a 90-deg deviation, the radii of the beam ($r_{i}$) before and after reflection on the ellipsoidal mirror are approximately equal. Under the paraxial approximation, the input and output NAs are given by ${\mathrm{NA}}_{i}=r_{i}/f_{i}$. The image space NA is, therefore, roughly equal to the fiber output NA multiplied by the angular magnification, $M_{\alpha }$, of the ellipsoidal mirror: $M_{\alpha }=f_{1}/f_{2}$. Although more complete descriptions of ellipsoidal mirror magnification (e.g., for high magnification or deviations different from 90 deg) can be found in the literature,^35^ the above approximation is sufficient in this context, as highlighted in the results section. The image space NA was chosen to achieve a sufficient depth of focus for OCT imaging, characterized by the Rayleigh length ($z_{R}$) of a Gaussian beam, and a target spot size, characterized by the beam waist ($w_{0}$). It is important to note that the single mode output divergence and corresponding NA of the DCF core are not well described by the specified fiber NA but rather by assuming a Gaussian beam propagation with a waist equal to the mode field diameter.^36^^,^^37^ For single-mode operation at 1300 nm in the DCF core, as is the case for OCT imaging, the fiber output NA is equal to 0.09 and not 0.12, as indicated in the spec sheet (DCF13, Thorlabs, Newton, New Jersey, United States). The second constraint is that the second focal length ($f_{2}$) be sufficiently long to enable the beam folding presented in Fig. 1(a), such that the focal plane is located ∼0.5  mm outside the outer surface of the capsule. This constraint arises from the size of the individual components, such as the ellipsoidal mirror, the micromotor, and the right-angle reflective prisms. The mechanical constraints are discussed further in the following section. Interestingly, $f_{2}$ may be adjusted by scaling $f_{1}$ accordingly to maintain the desired angular magnification. However, extending $f_{1}$ increases the distance between the fiber tip and the EM, which results in a longer total stiff length of the device. Finally, the third constraint is on the beam size at various positions along the beam path. Beyond a specific diameter, the beam will begin to clip on the housing, resulting in reduced beam quality and transmission efficiency. The limiting factor here is the beam size of the multimode output of the inner cladding of the DCF, which has a larger NA than that of the core: 0.2 for DCF13. This constraint applies when the inner cladding is used for both illumination and collection.

For esophageal imaging, we selected an image space NA of 0.02, resulting in a theoretical spot size of $w_{0}=21\;\mu m$ and a Rayleigh length of $z_{R}=1.04\;\mathrm{mm}$ at 1300 nm. For an SM core output NA of 0.09, we used the combination of $f_{1}=6.5\;\mathrm{mm}$ and $f_{2}=30\;\mathrm{mm}$ with a 90-deg deviation to achieve 0.0195 image space NA. This resulted in a maximum beam diameter at the EM of 2.6 mm from the DCF inner cladding. All parameters of the ellipsoidal surface were derived from the target values of $f_{1}$ and $f_{2}$ and Eqs. (1)–(3). These parameters were then used to diamond-turn 4-mm-diameter custom EMs out of aluminum (B-Con Engineering, Nepean, Canada). The subsequent beam folding was carried out using aluminum-coated right-angle prisms (Sumipro, Almelo, The Netherlands). The first prism after the EM had a 3-mm side length, whereas all others were 2-mm prisms. Finally, the DCF fiber was mounted into a 0.9-mm ceramic ferrule with a standard FC/APC 8-deg angle polished tip (Diamond SA, Losone, Switzerland). The DCF patch cord was terminated with an E2000 connector compatible with clinical sterilization protocols.

### 2.2 Mechanical Design

The RTCE is composed of a number of parts, as illustrated in the exploded view in Fig. 1(b). The first, colored in gray and located at the top of the image, houses the fiber ferrule. The outside is threaded to enable fine adjustment of the fiber holder’s axial position relative to EM by screwing it in and out of the second housing, shown in light blue in the diagram. A nut locks the ferrule holder in place once the optimal position is found. The yellow component is the EM holder. The EM is held in the central slot and can be locked in place using the screws visible next to the holder in the exploded view. A positioning groove was milled along the circumference of the EM at a known distance from the mirror base, allowing the EM to position itself axially as the screws are tightened. These screws are also used to perform fine adjustments of the EM position and orientation (tip and tilt), as described in the section on the alignment of the ellipsoidal mirror. The semi-transparent gray part below the yellow EM holder is the housing for the 2.4-mm-diameter hollow shaft micromotor (DBL024-05XX, Namiki Adamant, Tokyo, Japan). Care was taken to leave several hundred microns between the bottom faces of the motor and the EM to allow the motor’s electrical drive wire to be bent without being crushed. The final prism reflecting the light toward the outside of the capsule (pink in the diagram) is first mounted on a 600-μm-diameter rod, which is then inserted and glued into the hollow shaft of the micromotor. This sub-assembly, including a hollow-shaft motor, was purposely selected to enable the separate alignment of the final prism, as detailed in Sec. 2.3.2. The separate alignment reduces the handling of the motor and its exposure to liquid glue, which could potentially damage it. Although a similar result could be achieved without a hollow shaft, it might result in a longer stiff length or difficult handling of small components. A high-strength, medical-grade, 1-mm-thick glass window (DURAN^®^, Technoglas, Noordwijkerhout, The Netherlands) allows the laser light to reach the sample. Preliminary testing of the assembled housing (glued metal housing + glass window) indicated that it could handle stresses far exceeding normal operating conditions for longitudinal and transversal compression as well as longitudinal tension. Two V-grooves were milled into the top and bottom notches over which the glass window is slotted. These grooves are filled with polymer O-rings, visible in white Fig. 1(c), to add resistance and keep the components from sliding apart prior to the final fixation with glue (LOCTITE^®^ AA-3921, Henkel Corporation, Stamford, Connecticut, United States). The final part, located at the bottom of the exploded view, is the capsule cap, which holds two 2-mm fold prisms/mirrors to reflect the laser beam up toward the prism fixated on the micromotor. Aside from the glass window, all structural parts of the TCE were milled from aluminum. Two through-holes were drilled into all structural parts in the plane perpendicular to the one containing the laser path to ensure the proper rotational alignment of all parts relative to each other by sliding straight rods through them (not shown in the diagram).

### 2.3 Assembly

Because of the off-axis nature of the optical assembly and the resulting sensitivity to proper alignment, it was necessary to develop several intermediate assembly and verification steps to ensure the proper operation of the optical system.

#### 2.3.1 Ellipsoidal mirror alignment

**Fig. 3:**
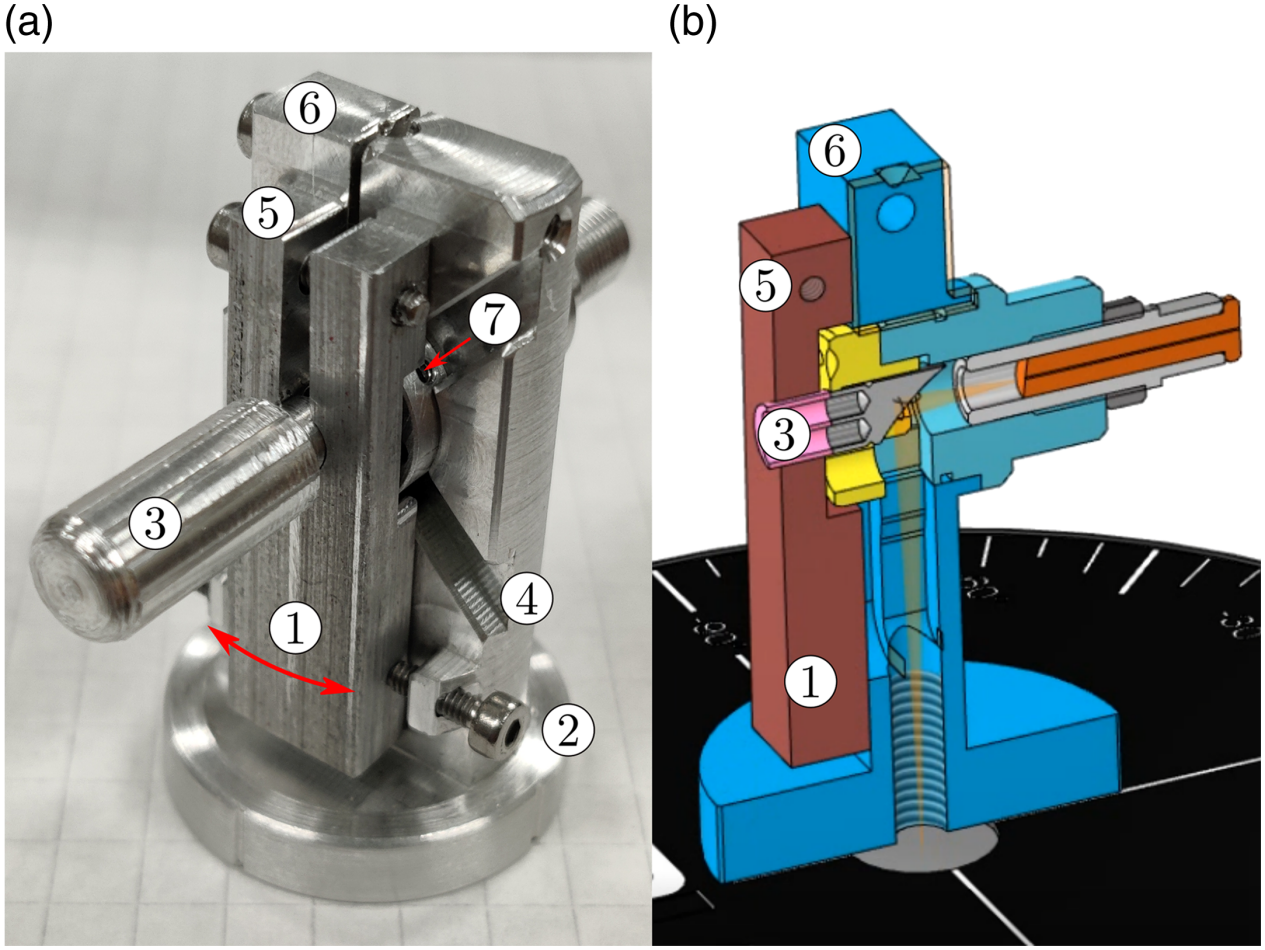
Alignment tool for the fine alignment of the ellipsoidal mirror. (a) Photograph of the tool. (b) Cross-section of the 3D model. (1) Clamp for fine rotational adjustments. (2) Adjustment screws to apply controlled rotation to the clamp. (3) Extension inserted into the EM for rotational adjustments. (4) Grooves to access tip and tilt screws. (5) Clamping mechanism for rotational adjustments. (6) Clamping mechanism for fiber and EM housings. (7) One of four tip and tilt adjustment screws.

The alignment of the EM and the fiber tip is critical to obtaining a diffraction-limited spot in the sample plane. Despite the precision manufacturing of the mirror, the mirror housing, and the fiber ferrule, some fine-tuning of the alignment remains necessary. To obtain real-time feedback, the fiber and EM subassembly were mounted onto a custom alignment tool, presented in Fig. 3. This subassembly includes all parts at or above the yellow EM housing in Fig. 1(b) except for the first beam folding prism (in brown). This tool is mounted onto a scanning-slit beam profiler (BP209IR1, Thorlabs) such that the theoretical focal plane ($f_{2}=30\;\mathrm{mm}$ here) is located precisely in the measurement plane of the beam profiler. This measurement plane is not the surface of the device but rather a few millimeters lower. The alignment tool is marked with centering lines that can be aligned with the beam profiler to ensure the proper orientation of the device. A small extension is inserted in the back of the EM and can be used for gross rotational alignment (3). Once completed, a clamp is fixed onto the extension (1 and 5), which can then be rotated using the adjustment screws at the extremity of the rotation lever (2). The fine adjustment screws for XY translation, tip, and tilt of the EM can be accessed via the diagonal grooves in the alignment tool (4 and 7). The EM slot in the housing is 200  μm wider than the outer diameter of the EM to allow for lateral translations and tip and tilt. The four fine alignment screws are positioned in two pairs facing each other and can be controlled individually for tip and tilt or in pairs for translation. In addition, the threaded housing of the fiber ferrule can be translated axially to adjust the distance between the fiber tip and the EM. All adjustment mechanisms were used iteratively to achieve the expected diffraction-limited spot size measured with the beam profiler. All optimization was performed at 1300 nm with the single-mode output of the DCF core. It is important to note that, although it is possible to achieve optimal alignment, the rudimentary nature of the alignment mechanisms does not guarantee a quick convergence nor the reversibility of the fine adjustment. As such, this process had to be repeated from the beginning several times to achieve the desired result. Once the assembly is in its final position, the tightened adjustment screws fixate the EM in place. The components’ positions are further stabilized by gluing the screws and the mirror to the housing.

#### 2.3.2 Motor shaft assembly

**Fig. 4:**
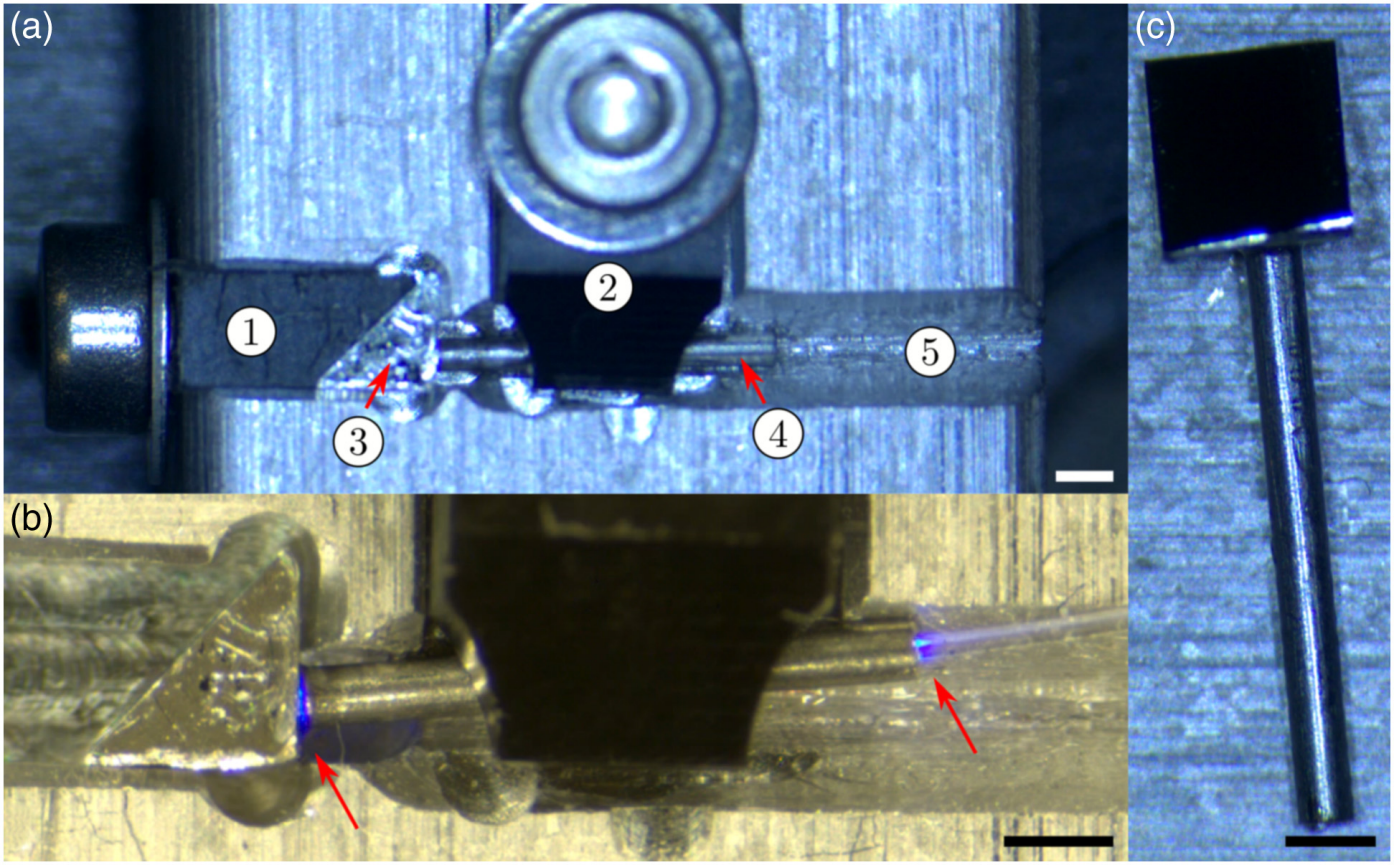
Motor shaft alignment tool and assembled prism and rod. (a) Loaded alignment tool with prism clamp (1), rod clamp (2), prism (3), hollow rod (4), and rod alignment groove (5). (b) Glued prism and rod assembly. Red arrows highlight the blue fluorescence of the UV-curing glue. (c) Final assembly to be inserted into the micromotor. All scale bars are 1 mm.

Another essential alignment is the orientation of the last prism, which deviates the laser beam toward the outside. The laser beam should not reach the transparent window under a normal incidence. Indeed, the air–glass and subsequent glass–air interfaces generate strong specular reflections that would decrease the signal-to-background ratio (SBR). A simple geometrical analysis shows that these specular reflections can be avoided if the incidence angle is greater than the half angle of the cone of the focused beam, i.e., $\theta_{\mathrm{inc}}>\sin^{-1}(\mathrm{NA})$. In this case, the largest collection NA is that of the inner cladding or NA = 0.04, so the angle of incidence must be greater than 2.5 deg. Assuming a perfectly vertical beam before the last prism, the incidence angle on the transparent window is equal to twice the tilt angle of the prism compared with it being perfectly aligned with the capsule’s vertical axis. As such, the prism must be tilted at least 1.25 deg. For good measure, we applied a 2-deg tilt to the prism, resulting in a 4-deg angle of incidence on the glass surface. This tilt was achieved using the mount presented in Fig. 4. An alignment groove (5) was milled into a holder to guide a hollow rod (4) to the back surface of a 2-mm reflective prism (3). Polymer clamps were three-dimensional (3D) printed to secure the components in place without damaging the optical surface of the prism (1 and 2). Before placement, the hollow rod was filled with ultraviolet (UV)-curing glue (LOCTITE AA-3921, Henkel Corporation, Stamford, Connecticut, United States). Once in place and secured, a UV-compatible multimode fiber was inserted into the rod until some glue could be observed flowing out from the rod/prism junction. The UV source injected into the fiber was then activated for several minutes until the curing was complete. Particular care was taken to avoid excessive overflow between the rod and the prism as this would hinder the proper insertion of the rod into the hollow shaft of the micromotor. The tool was designed to align the rod with the center of mass of the prism to minimize vibrations and radial shifts during rotations. The rod and prism assembly were then inserted into the motor’s hollow shaft and glued in place.

#### 2.3.3 Focal plane adjustment

**Fig. 5:**
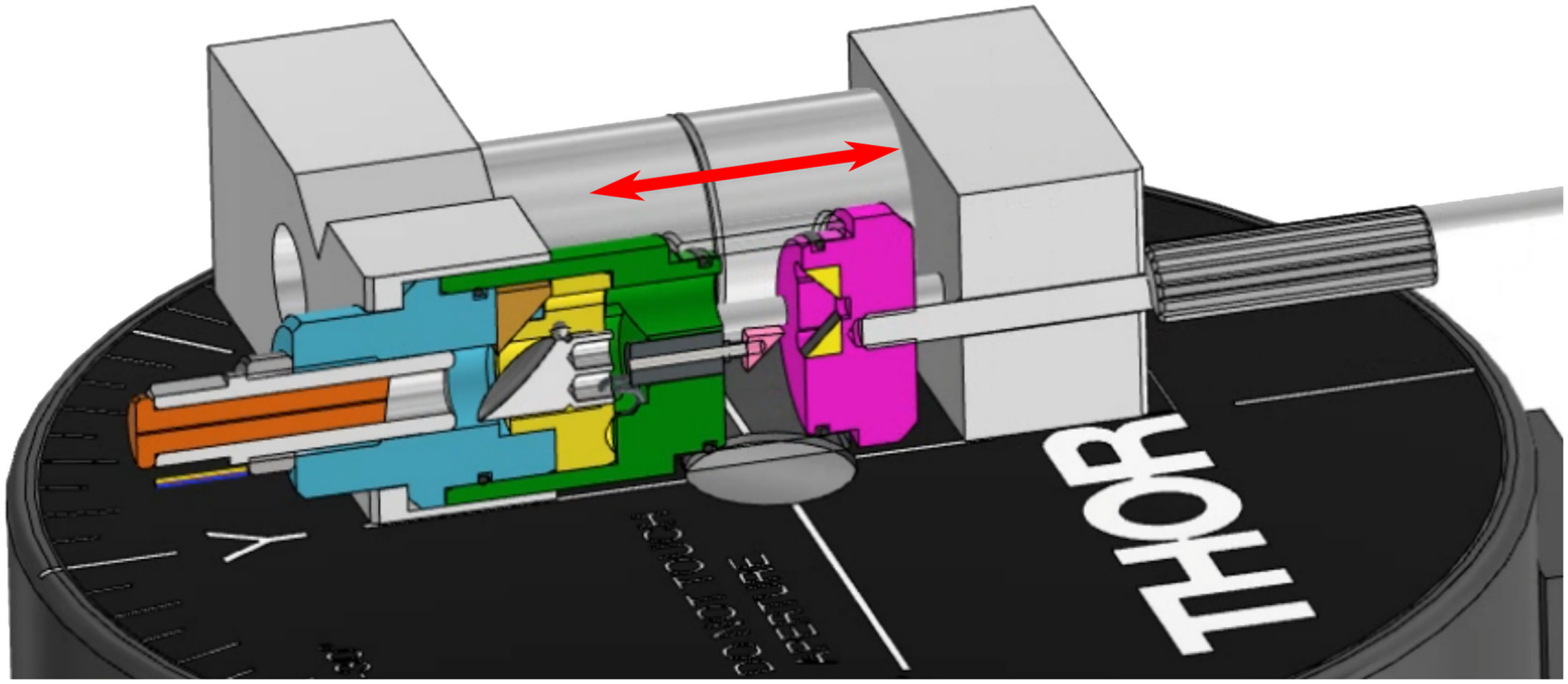
Focal plane alignment tool. The capsule cap is translated toward or away from the capsule body using the screw on the right.

The distance between the focal plane and the EM is fixed and defined by the surface parameters. However, the position of the focal plane relative to the outer surface of the TCE can be adjusted by extending the vertical beam path after the first fold prism [see Fig. 1(a)]. This can be achieved by slightly pulling the motor housing and cap apart, so long as the glass window remains securely on the notches. A final alignment tool was developed for this purpose and is depicted in Fig. 5. This tool is placed flat on the beam profiler and measures the beam diameter as the cap is moved closer or further away from the capsule body. By comparing the measured beam size to the prior beam profiling, it is possible to extrapolate the exact position of the focus relative to the measurement plane and the capsule surface. Once the desired focus position is achieved, the glass window and cap can be fixed into place by curing the glue applied prior to assembly. The O-rings also have the additional function of preventing glue overflow into the RTCE.

### 2.4 Complete Imaging System

**Fig. 6:**
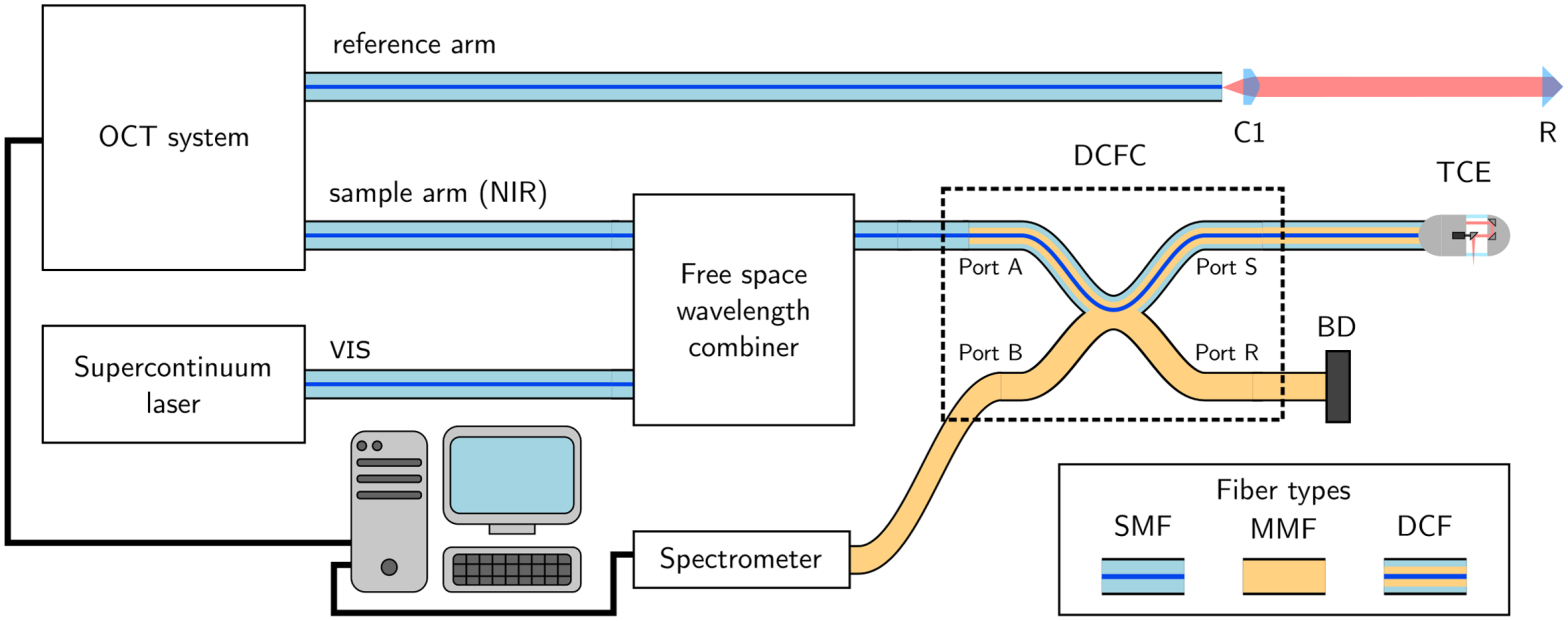
Complete experimental setup. C1, collimator; R, retroreflector; DCFC, double-clad fiber coupler; TCE, tethered capsule endoscope; BD, beam dump; SMF, single-mode fiber; MMF, multimode fiber; and DCF: double-clad fiber.

We used the fiber system presented in Fig. 6 for imaging. This system comprises a standard, 50-kHz swept-source OCT system at 1300 nm (system described in Ref. 38), with a free space wavelength combiner and a DCFC (DC1300LQ2, Castor Optics, Montreal, Canada) inserted in the sample arm. The wavelength combiner allows for the combination of OCT and VIS light in the core of an SMF28 fiber, which is then spliced to the DCF. The VIS light covers 450 to 1100 nm obtained by filtering (NKT Split, NKT Photonics Ltd., Southampton, United Kingdom) the output of a supercontinuum laser (EXU6, NKT Photonics Ltd.). Reflective collimators (RC04APC-P01, Thorlabs) were used in the beam combiner to accommodate the wide spectral range. Core illumination is used for both modalities, whereas collection is performed with the core for OCT and the inner cladding for reflectance imaging. The high-speed spectrometer (Cobra VIS CS550-600/200, Wasatch Photonics, Logan, Utah, United States) is used to collect spectroscopic information over the 500- to 700-nm spectral range. The two acquisition schemes are synchronized to the A-line trigger of the OCT system. Standard refractive optics were used for the OCT reference arm, resulting in a dispersion mismatch as the sample arm only includes reflective components. Numerical dispersion compensation was performed following a previously reported method^38^ to account for this. In this particular setup, it is straightforward to build a free-space reference arm because it must also compensate for the free-space segment in the wavelength combiner. However, of this free-space wavelength combiner is replaced with a fiber-based alternative (e.g., a fiber wavelength division/multiplexer), the only remaining free-space propagation in the sample arm would be that in the RTCE, equivalent to 36.5 mm (single pass). Constructing a reference arm with such a small free-space segment may prove challenging and add additional constraints on fiber length tolerances.

## 3 Results

### 3.1 Optical Performance

**Fig. 7:**
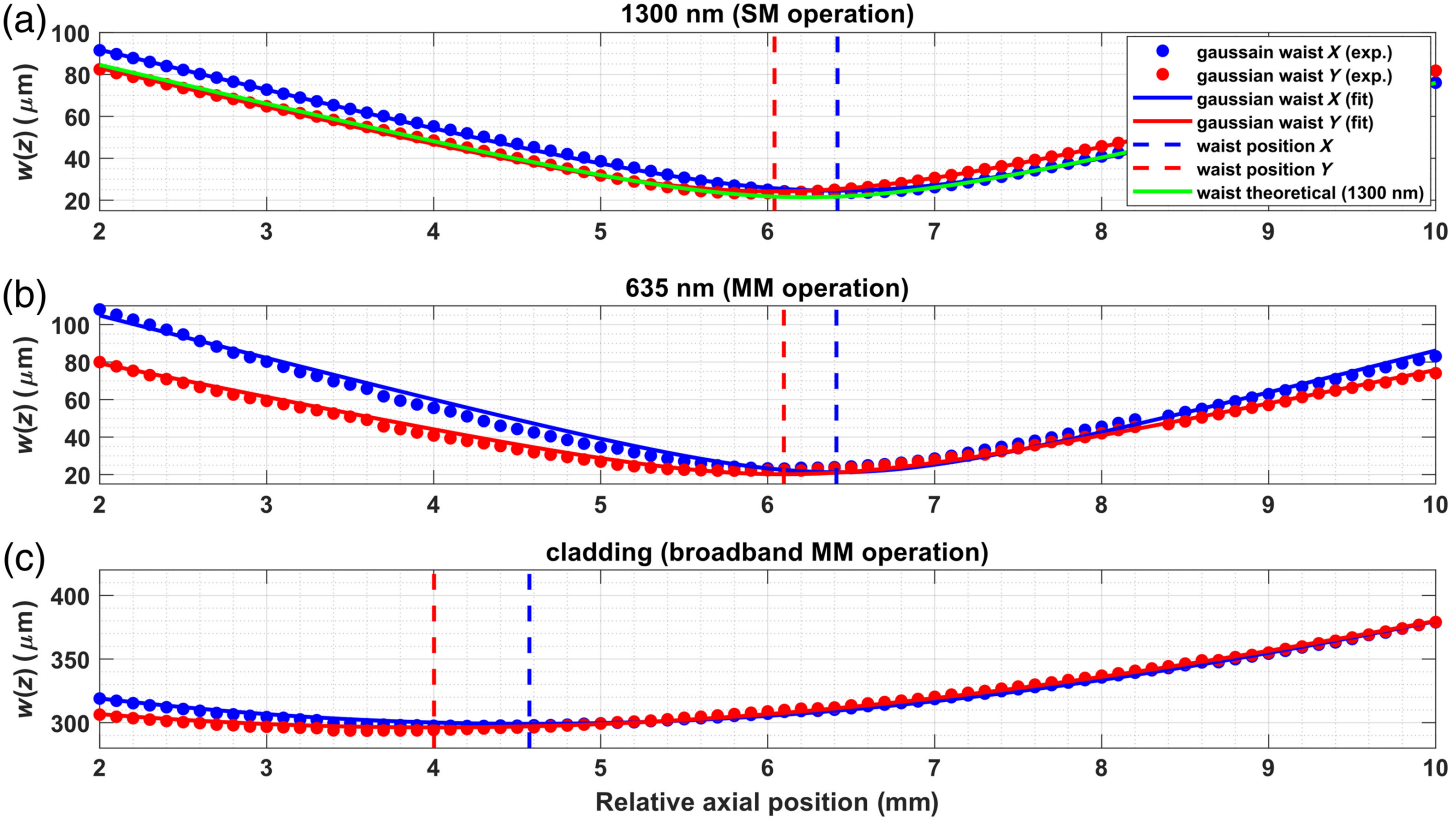
Beam profiling results for different illumination schemes. (a) Single-mode illumination at 1300 nm through the DCF core. (b) Multimode illumination at 635 nm through the DFC core. (c) Multimode broadband illumination (400 to 900 nm) through the DCF inner cladding.

We first characterized the capsule’s optical performance in terms of beam quality and overall transmission efficiency over a broad spectrum. Beam characterization was performed with the aligned EM before assembling the full capsule to maintain access to positions before and after the focal plane. As such, the characterization does not incorporate the effect of all optical components after the EM. However, the flat prism mirrors should not induce any aberrations, and the influence of the glass window was found to be minor in Zemax simulations (Zemax OpticsStudio, Canonsburg, Pennsylvania, United States). This influence includes a negligible alteration to the spot size, with the focal plane being shifted back by ∼500  μm, inducing some minor astigmatism. As such, the measured beam profiles should be representative of the beam at the output of the TCE. The output beam profiles, presented in Fig. 7, were measured from the core at 635 and 1300 nm and from the inner cladding using the broadband spectrum of the supercontinuum source. The beam profile was fitted to $w(z)=w_{0}\sqrt{1-(\frac{z-z_{f}}{z_{R}}{)}^{2}}$, where $w_{0}$ is the beam waist, $z_{f}$ is the axial position of the waist, and $z_{R}$ is the Rayleigh range. The extracted beam parameters are summarized in Table 1. It should be noted that the axial positions reported in Fig. 7 and Table 1 are relative values that correspond to the position of the translation stage used in the measurement. However, the focal plane for core illumination is indeed located at $f_{2}=30\;\mathrm{mm}$, guaranteed by the use of the alignment assembly presented in Fig. 3 and described in Sec. 2.3.1. The extracted beam parameters were determined from fitting experimental data points, where $w_{0}$ and $z_{R}$ were fitted independently. The theoretical values were calculated assuming NA = 0.0195. The difference in waist size and slight astigmatism (i.e., XY focal shift) makes it apparent that the final assembly is close to but not quite at the optimal alignment. However, the 15% increase in spot size compared with the theoretical value will not significantly degrade imaging quality, and the ∼400  μm shift between the X and Y focus should not be noticeable given the long Rayleigh range. As such, these values were considered adequate for practical applications. The near identical focal plane positions for 635 and 1300 nm depicted in Fig. 7 and reported in Table 1 demonstrate the expected achromatic behavior of the reflective components. This further highlights the RTCE’s potential ability to support a broad range of imaging modalities. The shift in the position of the beam waist for cladding illumination compared with core illumination is a known effect, described in detail by Beaudette et al.^39^ The spot size obtained with cladding illumination also represents the collection area when the cladding is used to capture light. Although the collection area varies with depth at the focal plane from core illumination, it is important to recognize that this variation is small across the Rayleigh range of the core illumination (where the sample should be located): <7% across the 5- to 7-mm range. As such, the collection area and corresponding collection efficiency should remain approximately constant in normal imaging conditions, i.e., when the sample is in contact with the outer surface of the RTCE.

**Table 1:** Beam parameters for different illumination schemes obtained from fitting.

	Waist (μm)		Focal plane pos. (mm)		Rayleigh range (mm)	
Illumination mode	$w_{0,x}$	$w_{0,y}$	$z_{f,x}$	$z_{f,y}$	$z_{R,x}$	$z_{R,y}$
Core 1300 nm (SM operation—theo.)	21.4		N/A		1.097	
Core 1300 nm (SM operation—exp.)	24.5	24.2	6.418	6.041	1.226	1.230
Core 635 nm (MM operation—exp.)	21.2	20.2	6.412	6.097	0.912	1.077
Cladding broadband (MM operation—exp.)	299.1	296.3	4.573	4.002	6.934	7.470

Theoretical values for core illumination calculated assuming image space NA = 0.0195. The reported Rayleigh range is a single-sided value.

**Fig. 8:**
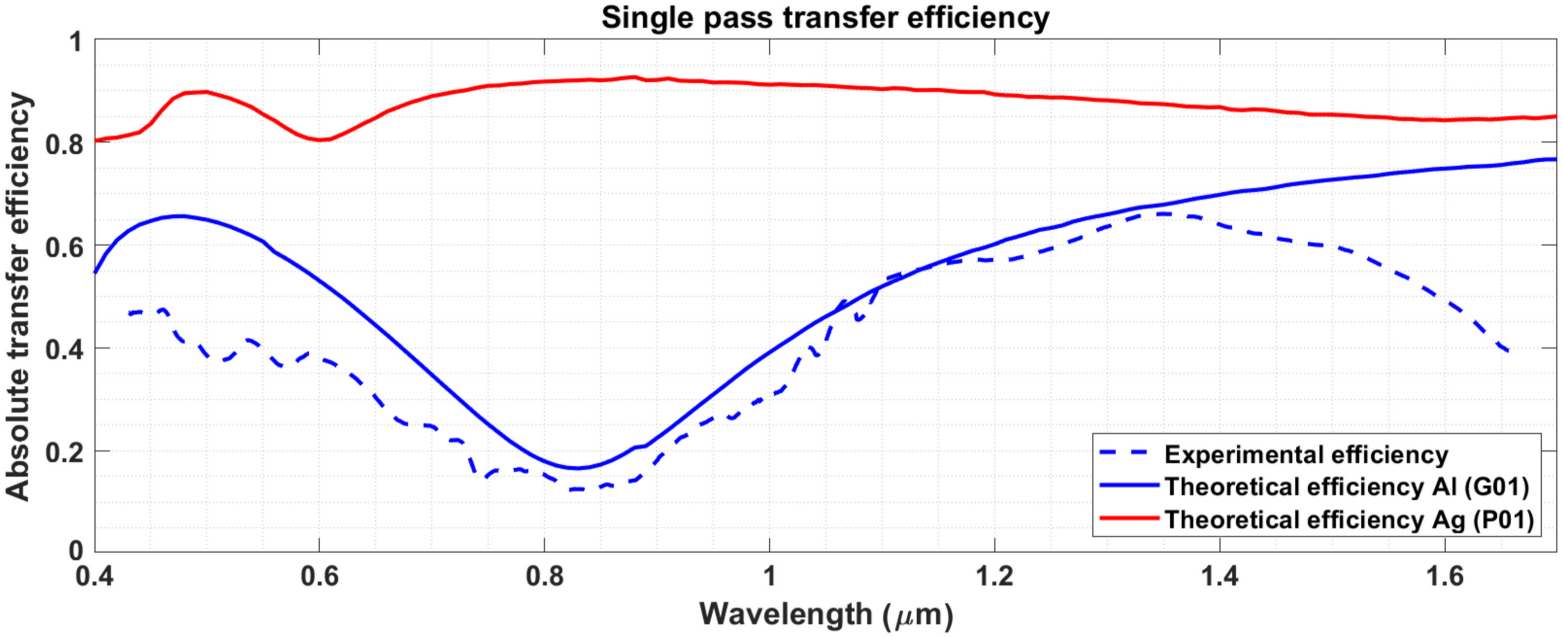
Single pass transfer efficiency. The theoretical efficiency curves were computed as $R^{5}(\lambda )$, where R is the reflectance of the different types of mirrors. Aluminum mirrors were used in this version of the TCE.

Figure 8 presents the transmission efficiency of the device across the VIS and NIR spectra, defined as the ratio of the input and output spectra. These spectra were measured by collecting the light before and after the capsule with a 600-μm fiber (NA=0.39) connected to either a VIS or NIR spectrometer (Avaspec-ULS2048CL-EVO and Avaspec-NIR256-EVO, Avantes, Apeldoorn, The Netherlands). To account for the NA-dependent transmission efficiency of the spectrometers, the spectra were normalized to absolute power measurements at 635 and 1300 nm for the VIS and NIR bands, respectively. The theoretical transmission curve was defined as the reflectance curve of protected aluminum (G01, Thorlabs) to the fifth power to account for all reflective elements in the beam path. It is apparent in Fig. 8 that the mirror reflectance is the principal source of loss. This results in very low efficiencies for specific wavelengths, which calls into question the choice of aluminum as a mirror surface. The problem is even further exacerbated by the fact that the presented curves must be squared to account for the double pass occurring during imaging. A more appropriate choice would be to use protected silver mirrors as they offer significantly better performance across all VIS/NIR wavelengths. For reference, the theoretical curve for protected silver (P01, Thorlabs) mirrors is also presented in Fig. 8. Mirror coatings from other manufacturers may differ slightly.

### 3.2 Preliminary Multimodal Imaging

**Fig. 9:**
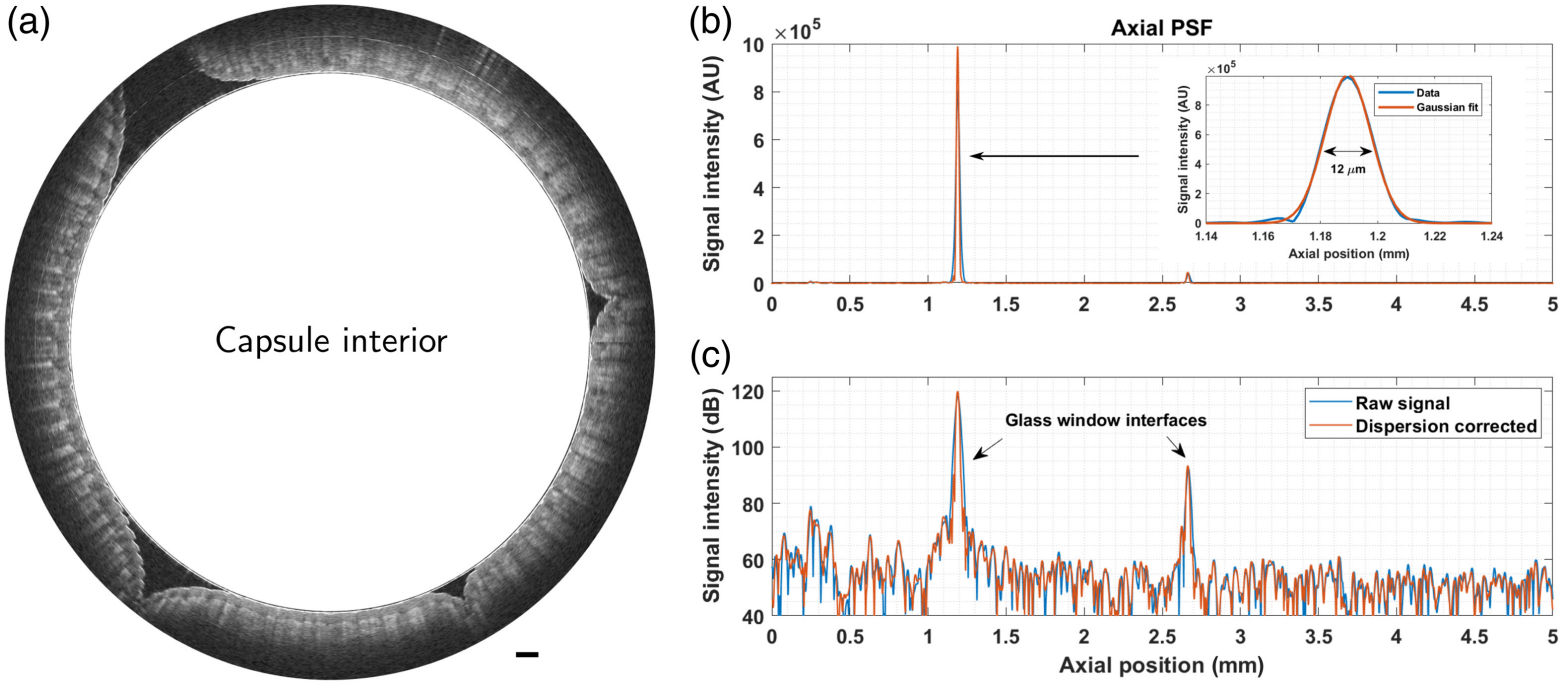
Preliminary OCT imaging. (a) B-scan of the fingers wrapped around the capsule. Capsule interior is not to scale (the center of the image does not correspond to the center of rotation), and the scale bar is equal to 1 mm. (b) Axial PSF obtained from reflections of the glass–air interfaces of the transparent window. The inset shows the Gaussian fit of the PSF. (c) Same as panel (b) but in a logarithmic scale (Video 1, MP4, 3.29 MB [URL: https://doi.org/10.1117/1.JBO.29.9.096003.s1]).

Figure 9(a) presents an OCT B-scan of fingers wrapped around the capsule, comprising 3300 A-lines acquired at a rotation speed of 940 rpm. This image highlights the achievable image quality and imaging depth (∼2  mm) despite the significant losses due to the double pass transmission through the endoscope and the free-space wavelength combiner. Figure 9(b) presents the axial point spread function (PSF) and Gaussian fit in linear scale, and panel (c) illustrates the impact of dispersion compensation in logarithmic scale. The axial resolution after k-linearization and numerical dispersion compensation [shown in Fig. 9(b)] was measured to be 12  μm. A video of an OCT acquisition of fingers wrapped around the RTCE is available in the Video 1.

**Fig. 10:**
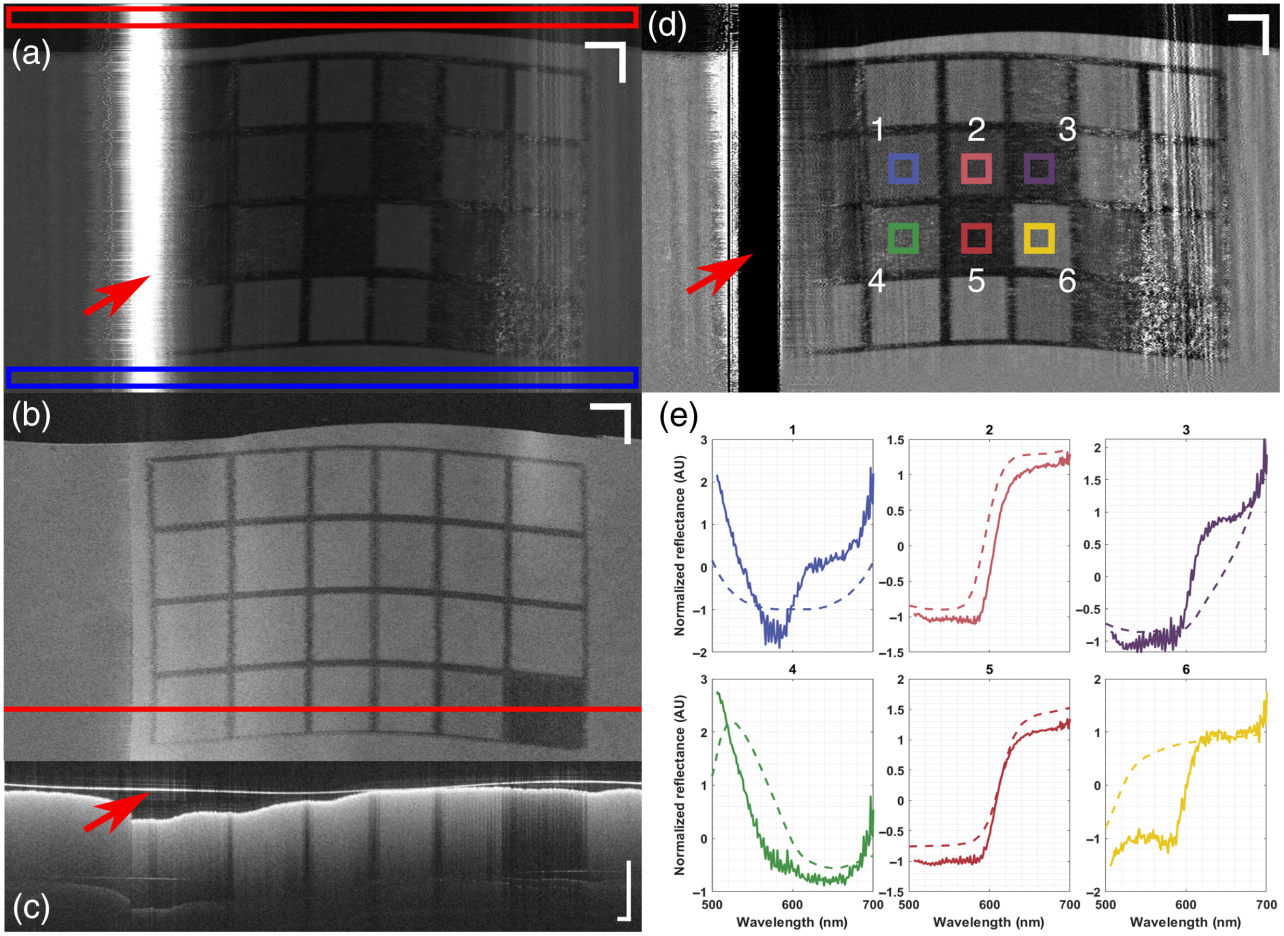
Combined imaging of a printed ColorChecker. (a) Raw image of the ColorChecker at 520 nm. The red box indicates the area of the image used as the dark measurement, where there was no sample around the capsule. The blue box indicates the part of the image used as the reference for calculating the reflectance spectrum. The red arrow highlights an area where strong specular reflections occur and the signal is saturated. (b) Matching en face OCT view. The red line shows the location of the cross-sectional view presented below. (c) OCT cross-section. The red arrow highlights the strong signal from the capsule’s external glass-air interface. (d) Reflectance image at 520 nm. The red arrow highlights the undesirable effect caused by the strong specular reflection, resulting in unrealistic reflectance values (below 0 or above 1). The intensity range was set to [0;2] to avoid saturation from pixels with glare. The colored squares numbered 1 to 6 are the ROIs used in the graphs below. (e) Spectral data averaged over the ROIs in panel (d). The solid lines are the experimental values, and the dashed lines are the theoretical values from the literature. All data were normalized by subtracting the mean and dividing by the standard deviation to compare spectral shapes effectively. Scale bars in panels (a), (b), and (d) are 3 mm and 1 mm in panel (c).

We also performed combined imaging OCT and reflectance spectroscopy. We imaged a printed ColorChecker target to demonstrate the reflectance mode imaging capabilities. The capsule was first fixed in place, and the ColorChecker was wrapped around it. The ColorChecker was then pulled back slowly using a motorized translation stage. The raw spectral image (i.e., the detected signal) at λ=520  nm is presented in Fig. 10(a). This image shows a white area where the signal is saturated (indicated by the red arrow) due to a strong specular reflection at one of the glass-air interfaces. The alignment of the beam and the non-perpendicular reflection occurring at the prism mounted on the micromotor should prevent this; however, the alignment of the final prism is likely incorrect. This can also be seen in the slight tilt in the red beam from the guide laser in Fig. 1(c). This saturation results in negative and invalid reflectance values, observable in Fig. 10(d). For each pixel, the reflectance was computed by dividing the dark-corrected signal from the sample by the dark-corrected signal from the reference. Here, a white section of paper was used as the reference, and the section with no sample was used as the dark signal, as indicated by the blue and red boxes in Fig. 10(a), respectively. Figures 10(b) and 10(c) present an en-face OCT image and a B-scan located at the red line in (B). Although no saturation occurs in these images, the strong reflection can be observed slightly in the en-face image and more clearly in (C), as indicated by the red arrow. Figure 10(d) presents the spectral reflectance image at λ=520  nm. The normalized reflectance spectra (solid lines) for each region of interest (ROI) highlighted in panel (d) are presented in Fig. 10(e) and compared with the theoretical values (dashed lines). It is apparent in Fig. 10(e) that the spectral trends between the experimental and theoretical values are similar but not identical. This mismatch may be attributed to differences in the reflectance spectrum of a printed ColorChecker compared with a real one. Nonetheless, Fig. 10 demonstrates the ability of the RTCE to perform co-registered imaging with both modalities, with all wavelengths in focus and most of the imaging range free of back reflections.

## 4 Discussion

### 4.1 Advantages and Limitations of the Proposed Design

The all-reflective design offers a variety of advantages over conventional designs relying on other focusing optics. First and foremost, it provides achromatic beam focusing, as demonstrated in Fig. 7. Perfectly achromatic beam focusing is valuable for multimodal imaging as well as for high-resolution OCT (typically referred to as μOCT), where axial variations of the illumination spectrum due to chromatic focal shifts can induce depth-dependent variations in the axial PSF and corresponding resolution.^40^^,^^41^ Previously proposed solutions have successfully corrected chromatic focal shifts for broadband and multimodal systems; however, these solutions often require advanced optical engineering and are specifically tailored to the utilized spectral ranges. In comparison, the proposed reflective design represents a one-size-fits-all solution. This design also removes optical interfaces producing unwanted back reflections, as reported in the existing literature.^28^^,^^42^^,^^43^

The proposed design also presents certain limitations. The first is the achievable numerical aperture in image space. Indeed, the image space NA is directly related to the fiber NA through the angular magnification of the EM. However, the angular magnification is strongly constrained by the device’s size. For example, the first focal length cannot be indefinitely extended to increase the magnification because it will extend the stiff length of the TCE. Moreover, extending $f_{1}$ will result in a larger beam size at the EM, requiring a larger clear aperture. However, for a fixed capsule diameter, there is a maximum beam and mirror size for which the beam is not clipped at the mirror or after the first fold prism. As such, the proposed design is ill-suited for techniques requiring a higher numerical aperture, such as multiphoton fluorescence microscopy or confocal microscopy.

A second shortcoming of the proposed design is the strict size constraint applied to the micromotor. Indeed, the motor must be as short as possible to minimize the overall length of the device and as narrow as possible to avoid beam-clipping. However, this leads to a very limited choice regarding suppliers and motor performance. Moreover, such miniature micromotors often lack feedback loops to inform of the angular position of the beam. As there is no electrical wire crossing the OCT field of view or other angular markers, it is difficult to assess any potential variations in rotation speed or other non-uniform rotational distortions (NURD). Such distortions can be observed in the video of fingers wrapped around the capsule, where the images appear slightly jittery (see Video 1). Although NURD is typically less significant with micromotor probes than with proximal scanning probes,^44^ some form of NURD correction is still necessary to optimize image quality or implement OCT angiography methods.^45^^,^^46^ Numerical methods using the correlation between overlapping A-lines could be used to this effect. However, such methods are computationally expensive and are difficult to implement in real time. Alternatively, markers could be added to the field of view at the expense of obscuring parts of the image. A second difficulty associated with the smaller motors is that they tend to operate at high minimum speeds, which increases the required A-line rate (or pixel rate for the non-OCT modalities) necessary to achieve Nyquist sampling along the circumference of the esophagus. Although modern OCT systems can reach MHz A-line rates, the additional modalities may require a lower imaging speed. Finally, the proposed opto-mechanical design is intrinsically more complex than most existing designs. Both the manufacturing of components and assembly of the various sub-systems are labor-intensive and sensitive to minute errors. Indeed, misalignments may lead to minor beam clipping and potential specular reflections off the glass window. These specular reflections and transfer losses can significantly reduce the signal-to-noise ratio (SNR) and SBR and degrade the overall image quality. Reduced the SBR is particularly problematic for reflectance spectroscopy as it renders the measurement more sensitive to spectral fluctuations of the source, leading to potentially significant errors in the calculated reflectance values.^47^^,^^48^ Such misalignments could be avoided by employing advanced micro-machining techniques that offer micron-scale tolerancing, such as micro-electrical discharge machining. These precise methods could facilitate more accurate manual alignment or eliminate the need for manual adjustments altogether. In addition, reducing the number of manipulations performed on optical components can help preserve their surfaces, thus preventing aberrations and maintaining high imaging quality.

### 4.2 Future Work

Future efforts will be centered around enhancing the device’s efficiency, improving the assembly process’s repeatability, and moving toward clinical certification for human use. Aside from replacing the aluminum mirrors with silver ones, the assembly process is also critical in the overall transfer efficiency. Further objectives will include validating all materials’ toxicology, stress testing the RTCE to establish failure mechanisms, and converting the optical system to a mobile clinical cart. We will also test the combination of OCT with a range of additional modalities, including multispectral fluorescence and reflectance imaging and dynamic laser marking.

## 5 Conclusion

We have demonstrated the feasibility of an all-reflective tethered capsule endoscope for esophageal imaging. The all-reflective design allows for achromatic and back reflection-free imaging across VIS and NIR wavelengths and could be utilized to combine various modalities with OCT through a double-clad fiber. Such a device could accelerate the clinical translation of multimodal OCT in the esophagus by avoiding iterative optical design and potential re-certification.

## Supplementary Material



## Data Availability

The datasets presented in this paper are available from the corresponding author upon reasonable request.
